# MetiTree: a web application to organize and process high-resolution multi-stage mass spectrometry metabolomics data

**DOI:** 10.1093/bioinformatics/bts486

**Published:** 2012-07-31

**Authors:** Miguel Rojas-Chertó, Michael van Vliet, Julio E. Peironcely, Ronnie van Doorn, Maarten Kooyman, Tim te Beek, Marc A. van Driel, Thomas Hankemeier, Theo Reijmers

**Affiliations:** ^1^Netherlands Metabolomics Centre, Einsteinweg 55, 2333 CC Leiden and ^2^Division of Analytical Biosciences, Leiden/Amsterdam Center for Drug Research, Leiden University, Einsteinweg 55, 2300 RA Leiden and ^3^Netherlands Bioinformatics Centre, Geert Grooteplein 28, 6525 GA Nijmegen and ^4^TNO Research Group Quality and Safety, P.O. Box 360, NL-3700 AJ Zeist, The Netherlands

## Abstract

**Summary:** Identification of metabolites using high-resolution multi-stage mass spectrometry (MS*^n^*) data is a significant challenge demanding access to all sorts of computational infrastructures. MetiTree is a user-friendly, web application dedicated to organize, process, share, visualize and compare MS*^n^* data. It integrates several features to export and visualize complex MS*^n^* data, facilitating the exploration and interpretation of metabolomics experiments. A dedicated spectral tree viewer allows the simultaneous presentation of three related types of MS*^n^* data, namely, the spectral data, the fragmentation tree and the fragmentation reactions. MetiTree stores the data in an internal database to enable searching for similar fragmentation trees and matching against other MS*^n^* data. As such MetiTree contains much functionality that will make the difficult task of identifying unknown metabolites much easier.

**Availability:** MetiTree is accessible at http://www.MetiTree.nl. The source code is available at https://github.com/NetherlandsMetabolomicsCentre/metitree/wiki.

**Contact:** m.rojas@lacdr.leidenuniv.nl or t.reijmers@lacdr.leidenuniv.nl

## 1 INTRODUCTION

Metabolite identification is a challenging but essential step for the interpretation and understanding of many biological processes for an increasing number of applications such as biomarker discovery, drug discovery or nutritional studies. The feasibility of using multi-stage mass spectrometry (MS*^n^*) for identification of metabolites has been shown before (Sheldon *et al.*, 2009). The complexity of the data generated demands new computational infrastructures to organize the data and to extract relevant information. Recently, databases have been set up for storing fragmentation spectra such as MS*^n^* data (e.g. MassBank; Horai *et al.*, 2010), and tools have been developed to process MS*^n^* data (e.g. the MEF tool; Rojas-Chertó *et al.*, 2011), or to compare MS*^n^* data (e.g. Mass Frontier; Thermo Fisher Scientific). In this article we present a web application called MetiTree with the novelty that it combines the processing of high-resolution MS*^n^* data with a personal local library to organize the fragmentation data. Furthermore, it allows the comparison of MS*^n^* data to help the researcher with the identification of metabolites. MetiTree is available at http://www.MetiTree.nl together with some test MS*^n^* data.

## 2 METHODS

### 2.1 Web application

MetiTree (Metabolite Identification Tree) is a web application intended to aid in the metabolite identification process. Currently, MetiTree offers the possibility to organize, process, share, visualize and search for similar high-resolution MS*^n^* data. MetiTree’s web interface is accessed through a web browser and it was created using the Grails (http://grails.org) frame-work.

### 2.2 Data processing and comparison

In order to process MS*^n^* data, MetiTree integrates the MEF tool (Rojas-Chertó *et al.*, 2011), which extracts chemical information from the fragments assigning the elemental composition to the ions and neutral losses. MetiTree also allows the comparison of newly acquired MS*^n^* data to data that are already stored in an internal library (Rojas-Chertó *et al.*, 2012).

### 2.3 Data visualization

MetiTree incorporates a JavaScript spectral tree viewer developed to visualize MS*^n^* data (https://trac.nbic.nl/brsp201017/), in order to facilitate the exploration, interpretation and validation of the results. This viewer interconnects three MS*^n^* items: the spectrum, which contains mass peaks, the fragmentation tree, which contains fragment nodes/elemental compositions, and the fragmentation reactions, which contain structures.

## 3 USAGE EXAMPLE

MS*^n^* data previously published by our group (Rojas-Chertó *et al.*, 2011, 2012) are used to demonstrate how metabolites can be identified using the MetiTree web application. These data are freely accessible as test data in MetiTree.

### 3.1 Data processing

The required input to process MS*^n^* data is mzXML files and the settings of the processing parameters. Processing parameters are grouped into those to extract the mass spectrometry information (*m*/*z*, intensity and retention time) and those to enrich the MS data with chemical information (elements and number of atoms). MetiTree allows individual file as well as batch processing. Furthermore, the same mzXML file can be processed several times with different sets of parameters. The results and parameters’ information are stored to allow for posterior revision.

### 3.2 Data visualization

**Fig. 1.**
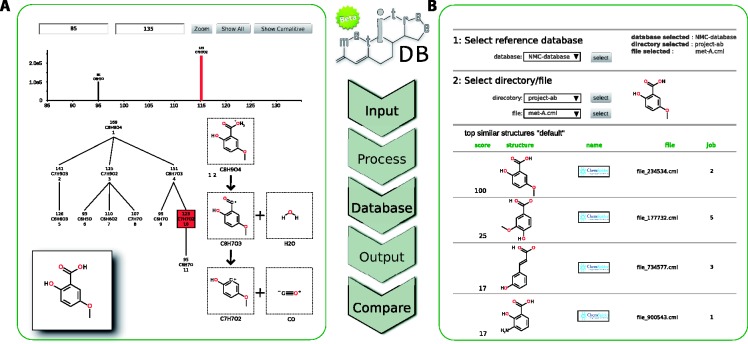
Overview of the MetiTree process flow. After the user submits MS*^n^* spectra, MetiTree will process these according to a set of parameters. Afterwards, the processed data can be stored in an internal library and labeled as a reference compound using the InChI identifier. The results are presented in different formats and viewers, which facilitates the exporting of text and figures for their use in reports and publications (**A**). Finally, MS*^n^* data can be queried to find similar MS*^n^* data in the library (**B**) and the query results are presented in a list

Once the data are processed, it can be displayed using the spectral tree viewer (Fig. 1A). When the node (a fragment) is selected, the corresponding spectrum is displayed together with the concatenated reactions that connect the parent ion with the selected fragment. The structure of the fragment can only be displayed if it has been previously assigned. The results generated by MetiTree can be exported to different formats (CSV, CML; Murray-Rust and Rzepa, 1999) and PDF) for further analysis or for presenting results in reports and publications.

### 3.3 Library storage

MetiTree creates directories for grouping mzXML files, assisting with the organization of the data according projects or topics. Processed MS*^n^* data can be stored in one or multiple internal databases (Fig. 1B). Because the users are organized in groups, they can share files and libraries with other group members. All MS*^n^* data can be labeled with an InChI identifier of the compound, which is automatically cross-referenced with PubChem and ChemSpider databases.

### 3.4 Data search

MetiTree integrates the functionality to query for similar MS*^n^* data (Rojas-Chertó *et al.*, 2012) stored in the library. The results are presented in a list showing the chemical structures of the most similar MS*^n^* data and the corresponding similarity values. A value near 100 indicates that MS*^n^* data are highly similar, while a value close to 0 illustrates that they are very different. If a fragmentation tree of the same compound is present in the library, complete identification is possible (identity search). If similar fragmentation data are found (similarity search), this substructure information can be used to generate candidate structures of the unknown compound (partial identification).

### 3.5 Future

In the near future this new web application will also accept the uploading of other types of MS*^n^* files (e.g. cml, mzML) and manual annotation of MS*^n^* data with chemical structural information (assigning substructures to the nodes of the fragmentation trees) will also be possible.

## 4 CONCLUSION

The growing interest in metabolite identification has increased the need to create computational and visual tools for MS*^n^* analysis. MetiTree, which gathers several in-house developed tools, is an easy-to-use web application that combines processing, sharing, visualizing and querying MS*^n^* data to help researches to identify metabolites of interest and decrease the time-consuming task of identifying metabolites.
